# Hemolacria secondary to major depressive disorder and generalized anxiety disorder: A case report

**DOI:** 10.1002/ccr3.8127

**Published:** 2023-11-09

**Authors:** Leila Razeghian Jahromi, Mehdi Ghaderian Jahromi, Mahsa Ghavipisheh, Iman Ahrari

**Affiliations:** ^1^ Substance Abuse and Mental Health Research Center Shiraz University of Medical Sciences Shiraz Iran; ^2^ Medical Imaging Research Center Shiraz University of Medical Sciences Shiraz Iran; ^3^ Research Center for Psychiatry and Behavior Science Shiraz University of Medical Sciences Shiraz Iran; ^4^ Neurosurgery Department Namazi Hospital, Shiraz University of Medical Sciences Shiraz Iran

**Keywords:** blood, generalized anxiety disorder, hemolacria, major depressive disorder, tears

## Abstract

**Key Clinical Message:**

Hemolacria can occur on the basis of a psychiatric disorder without an organic cause. However, this should be a diagnosis of exclusion. Treatment of the underlying psychiatric illness may relieve this condition.

**Abstract:**

A 24‐year‐old man presented with the chief complaint of bloody tears, which began 4 months earlier after commencing mandatory military service. He had no underlying diseases, and all work‐ups returned normal, though a microscopic examination confirmed red blood cells. He was diagnosed with hemolacria secondary to generalized anxiety disorder and major depressive disorder, responding to propranolol and sertraline. Hemolacria was totally cured after 6 months of treating the underlying psychiatric illness.

## 1 INTRODUCTION

Some rare medical conditions can remarkably affect a patient's quality of life. One of these conditions is a rare clinical phenomenon in which blood is present in body secretions. Although such secretions could occur secondary to trauma, cancer, infection, bleeding diathesis, inflammation, or hypertensive crisis,^1^ some patients have bloody secretions without a known organic cause. The bloody secretion could be from sweat glands,^2^ eyes,^3^ or ears,^4^ called hematohidrosis, hemolacria, and blood otorrhea, respectively. Experiencing hematohidrosis, hemolacria, or blood otorrhea merely due to a psychiatric disorder is a rare clinical condition.^5^, ^6^

## 2 CASE REPORT

**FIGURE 1:**
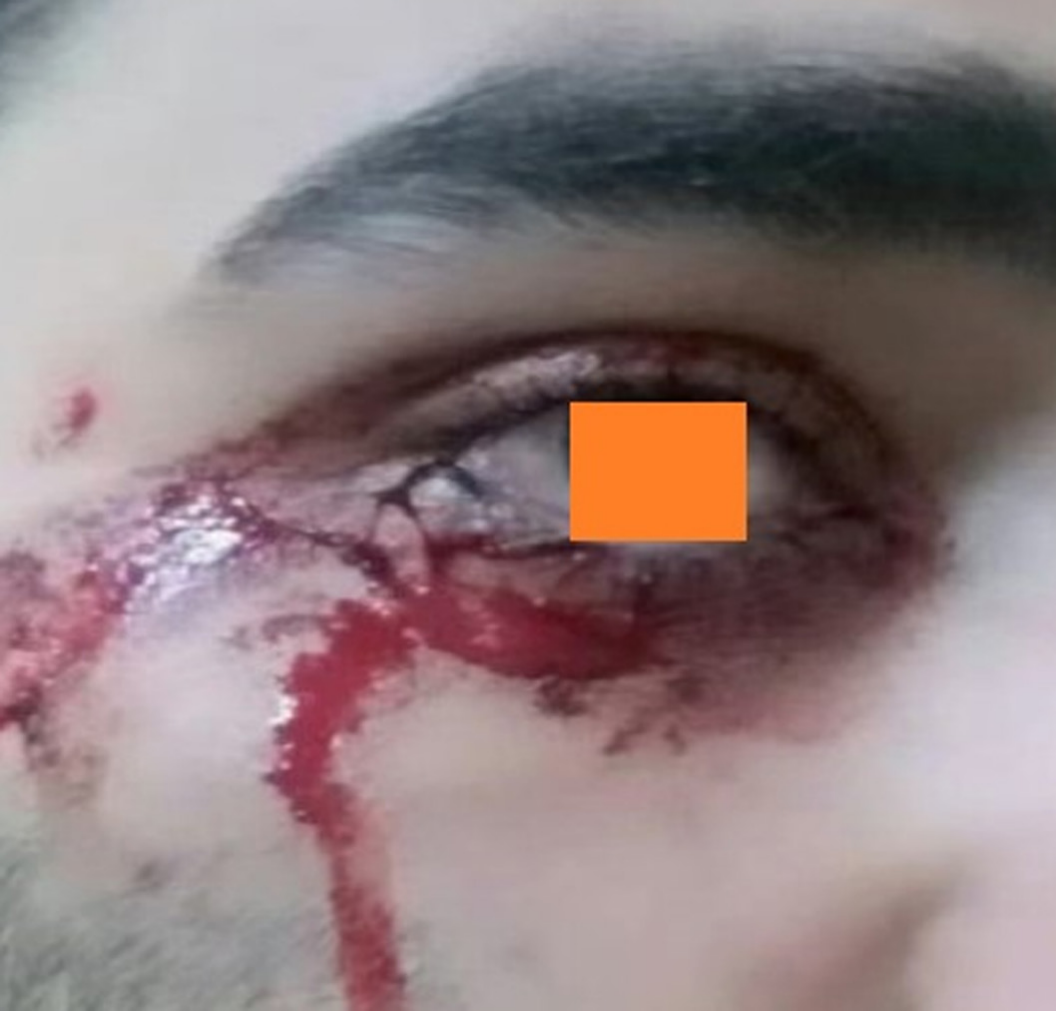
Clinical picture of the patient showing hemolacria at presentation.

A 24‐year‐old single man from Shiraz, Iran, referred to an ophthalmologist due to bloody tears in his eyes for about 4 months (Figure 1). The bloody tears started after great distress but were not secondary to tears of joy. The patient had a bachelor's degree and was born in Shiraz. He was raised in a high socioeconomic family and lived with his parents. Medical history of hematologic disorders was unremarkable in the patient and his family. Also, he did not report a history of infection, inflammation, malignancy, trauma, contact with chemicals, or taking medication since his problem had started. Besides, a physical examination of the eyes, lacrimal glands, and facial skin was normal. All requested lab data were normal, including complete blood count, prothrombin time, partial thromboplastin time, thrombin time, and bleeding time. Pathologic evaluation of bloody tears under the light microscope showed red blood cells, confirming the diagnosis of hemolacria. Furthermore, the eye cavity and sinuses were reported normal on the computerized tomography scan of the sinuses and lacrimal glands. On the ocular sonography, the anterior and posterior chambers and visualized parts of periocular structures were normal in appearance, with no sign of a definite pathology bilaterally.

Considering the normal physical examination and paraclinical data, the patient was referred to a psychiatrist as he had signs and symptoms of severe depression and anxiety. A comprehensive history taken by the psychiatrist revealed that bloody tears started 4 months after the patient had been recruited for mandatory military service. However, the patient did not request an exemption from continuing military service, ruling out malingering. The patient cried during the interview, and the bloody tears were directly observed, ruling out a factitious disorder. His affect was depressed. The patient had restlessness, irritability, and sleep disturbance. He fatigued easily and showed a lack of concentration in the mental examination. He also had no family history of psychiatric disorders. Ultimately, the mental examination resulted in the diagnosis of generalized anxiety disorder and major depressive disorder. Hence, propranolol 20 mg/day and sertraline 50 mg/day were prescribed for the patient, and he was visited weekly for the first month of follow‐up. Based on the patient's report, the frequency and severity of hemolacria remarkably decreased 2 weeks after treatment commencement and completely disappeared at the end of Week 4. As the depressive signs and symptoms persisted, the treatment continued for 6 months, although tapering was started after 4 months. Interestingly, he had no symptoms in the follow‐ups, which continued up to 6 months after treatment discontinuation.

## 3 DISCUSSION

The present case report indicates a relationship between hemolacria and psychiatric disorders. As mentioned, the patient was a young educated man from a supportive family with high socioeconomic status. To explain his idiopathic bloody secretion, it can be hypothesized that great distress resulted in the enormous release of catecholamine neurotransmitters, causing remarkable blood vessel constriction. After relief from distress, the blood vessels would dilate significantly, which could cause a rupture of the capillaries.^7^, ^8^ Another theory is that chronic stressful situations, including depressive disorders, would cause great changes in the immune and endocrine systems.^9^ One of the changes is a remarkable increase in blood cortisol levels, which would significantly alter cardiovascular system function, increasing capillary permeability. Therefore, one of the results could be the distressing phenomenon known as a bloody secretion.^9^

The present case indicates that pure hemolacria can occur following major psychiatric disorders, which should be considered only after ruling out other possible diagnoses that could result in bloody tears. Our report implicates that in the case of anxiety and depression, treating the patient with a beta‐blocker and selective serotonin‐reuptake inhibitor is effective in relieving the patient's bloody secretion. The strengths of this case report were the complete work‐up to rule out our other causes of hemolacria. However, the weakness of this report was that long‐term follow‐up was not accomplished. Overall, it seems necessary to conduct a large multicentric case–control study to identify the determinant factors of hemolacria in association with psychiatric disorders. Also, physicians should remember that a rare phenomenon such as hemolacria could have a psychiatric basis.

## AUTHOR CONTRIBUTIONS

**Leila Razeghian Jahromi:** Conceptualization; data curation; investigation; writing – original draft; writing – review and editing. **Mehdi Ghaderian Jahromi:** Conceptualization; investigation; writing – original draft; writing – review and editing. **Mahsa Ghavipisheh:** Investigation; project administration; writing – original draft; writing – review and editing. **Iman Ahrari:** Data curation; formal analysis; writing – original draft; writing – review and editing.

## FUNDING INFORMATION

None.

## CONFLICT OF INTEREST STATEMENT

The authors have no conflict of interest to declare.

## CONSENT

Written informed consent was obtained from the patient to publish this report in accordance with the journal's patient consent policy.

## Data Availability

All data are included in the manuscript.
